# Poster Session I - A166 STOOL HEMOGLOBIN OF OLDER PATIENT POLYP SURVEILLANCE (SHOPPS): FEASIBILITY OF FIT COLLECTION AND INTERIM PROGRESS REPORT

**DOI:** 10.1093/jcag/gwaf042.166

**Published:** 2026-02-13

**Authors:** C Tai, M Sey, B Yan, T Ponich, N Khanna, J Gregor

**Affiliations:** Gastroenterology, Western University Schulich School of Medicine & Dentistry, London, ON, Canada; Gastroenterology, Western University Schulich School of Medicine & Dentistry, London, ON, Canada; Gastroenterology, Western University Schulich School of Medicine & Dentistry, London, ON, Canada; Gastroenterology, Western University Schulich School of Medicine & Dentistry, London, ON, Canada; Gastroenterology, Western University Schulich School of Medicine & Dentistry, London, ON, Canada; Gastroenterology, Western University Schulich School of Medicine & Dentistry, London, ON, Canada

## Abstract

**Background:**

Colorectal cancer (CRC) is a leading cause of cancer-related death in older adults, particularly those with a history of colon polyps. However, surveillance guidance beyond age 75 is lacking. As a result, many individuals aged 75–84 with prior polyps continue to undergo colonoscopies despite uncertain benefit. The Fecal Immunochemical Test (FIT) may provide a safer alternative for surveillance in this population. Determining whether FIT can reliably exclude advanced neoplasia could reduce unnecessary colonoscopies and optimize the use of endoscopy resources.

**Aims:**

This interim report evaluates the feasibility of FIT kit collection among older adults undergoing surveillance colonoscopy and provides preliminary data on the negative predictive value (NPV) of FIT for high-risk polyps (HRP) or CRC in this population.

**Methods:**

This study is a prospective study aiming to recruit 417 patients aged 75–84 with a history of prior polypectomy who are already scheduled for surveillance colonoscopy at London Health Sciences Centre and St. Joseph’s Health Care London. Participants are asked to complete a FIT prior to their scheduled colonoscopy. FIT results do not influence clinical care, and all participants undergo colonoscopy as planned. The primary outcome is the NPV of FIT for detecting CRC or HRP, defined as polyps ≥10 mm, ≥3 adenomas, tubulovillous adenoma, high-grade dysplasia, or sessile serrated polyps.

**Results:**

To date, 82 patients have been recruited, of whom 1 has passed away. The FIT kit return rate was 69.1% (56/81), with 6.2% testing positive (5/81). Among these 56 participants, 38 have completed colonoscopies with pathology results available; none were found to have CRC. Of these 38 participants, 30 had negative FIT results with no HRP identified on colonoscopy, while 5 had negative FIT results but were found to have HRP, yielding a preliminary NPV of 85.7% for FIT in detecting HRP. One participant had a positive FIT with HRP confirmed, and two had positive FIT results without HRP on colonoscopy. Stratifying by history, 14 participants had a history of HRP and 24 had a history of low-risk polyps (LRP). Among those with prior HRP, the NPV was 72.7% (8 with FIT-/HRP- and 3 with FIT-/HRP+). Among those with prior LRP, the NPV was 90.9% (20 with FIT-/HRP- and 2 with FIT-/HRP+). No serious adverse events have been reported to date.

**Conclusions:**

Preliminary data demonstrate that FIT kit collection is feasible in older adults undergoing surveillance colonoscopy and suggest a high NPV of FIT, particularly among those with a history of low-risk polyps. These findings support FIT as a reasonable alternative to colonoscopy for guiding surveillance in this population. Continued recruitment will allow for more precise estimates and further assessment of its clinical implementation.

**Funding Agencies:**

None

